# Application of a six sigma model to evaluate the analytical performance of urinary biochemical analytes and design a risk‐based statistical quality control strategy for these assays: A multicenter study

**DOI:** 10.1002/jcla.24059

**Published:** 2021-10-15

**Authors:** Qian Liu, Guangrong Bian, Xinkuan Chen, Jingjing Han, Ying Chen, Menglin Wang, Fumeng Yang

**Affiliations:** ^1^ Department of Laboratory Medicine The Second People's Hospital of Lianyungang Lianyungang China; ^2^ Department of Laboratory Medicine Xuzhou Medical University Affiliated Hospital of Lianyungang Lianyungang China; ^3^ Department of Laboratory Medicine Wuxi Branch of Ruijin Hospital Wuxi China; ^4^ Department of Laboratory Medicine Nantong Hospital of Traditional Chinese Medicine Nantong China; ^5^ Department of Laboratory Medicine Suqian First Hospital Suqian China

**Keywords:** analytical performance, quality goal index, risk‐based statistical quality control strategy, six sigma, urinary biochemical analytes

## Abstract

**Background:**

The six sigma model has been widely used in clinical laboratory quality management. In this study, we first applied the six sigma model to (a) evaluate the analytical performance of urinary biochemical analytes across five laboratories, (b) design risk‐based statistical quality control (SQC) strategies, and (c) formulate improvement measures for each of the analytes when needed.

**Methods:**

Internal quality control (IQC) and external quality assessment (EQA) data for urinary biochemical analytes were collected from five laboratories, and the sigma value of each analyte was calculated based on coefficients of variation, bias, and total allowable error (TEa). Normalized sigma method decision charts for these urinary biochemical analytes were then generated. Risk‐based SQC strategies and improvement measures were formulated for each laboratory according to the flowchart of Westgard sigma rules, including run sizes and the quality goal index (QGI).

**Results:**

Sigma values of urinary biochemical analytes were significantly different at different quality control levels. Although identical detection platforms with matching reagents were used, differences in these analytes were also observed between laboratories. Risk‐based SQC strategies for urinary biochemical analytes were formulated based on the flowchart of Westgard sigma rules, including run size and analytical performance. Appropriate improvement measures were implemented for urinary biochemical analytes with analytical performance lower than six sigma according to the QGI calculation.

**Conclusions:**

In multilocation laboratory systems, a six sigma model is an excellent quality management tool and can quantitatively evaluate analytical performance and guide risk‐based SQC strategy development and improvement measure implementation.

## Graphical abstract

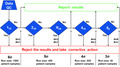

## 1 INTRODUCTION

Urinary quantitative biochemical analytes mainly include potassium (K), sodium (Na), chloride (Cl), calcium (Ca), phosphorus (P), glucose (GLU), urea, creatinine (Crea), total protein (TP), and microalbumin (mALB), and their clinical applications are becoming increasingly widespread.^1^, ^2^, ^3^, ^4^, ^5^ The levels of K, Na, Cl, Ca, and P reflect the excretion and reabsorption functions of the kidneys.^6^, ^7^ The detection of GLU levels is mainly used for the auxiliary diagnosis of diabetes.^8^ The levels of urea, Crea, TP, and mALB mainly reflect the degree of kidney damage caused by various diseases.^9^, ^10^, ^11^ With the widespread application of urinary biochemical analytes in clinics, the testing capabilities of laboratories are increasingly becoming a challenge. Therefore, laboratories urgently need to design a quality evaluation strategy to evaluate the analytical performance of urinary biochemical analytes.

As an important quality management tool, the six sigma model was first introduced to clinical laboratories by Nevalainen et al.^12^ to evaluate the performance of an analytical process. As an important parameter for evaluating the analytical performance of laboratories, the sigma metric has a significant advantage in quantitative evaluation.^13^ Once the analytical performance of the laboratory achieves six sigma, there are only 3.4 errors per one million test results (the defect rate per million is 3.4), and the detection capability of the laboratory has reached the "world‐class" level.^14^ The six sigma model is mainly composed of three variables: the total allowable error (TEa), bias, and coefficient of variation (CV). Bias reflects the trueness of analytes, and CV reflects the imprecision of analytes, both of which represent the analytical performance of the laboratory analytical system. However, TEa is closely related to the quality goal selected by the laboratory and is not directly related to the analytical performance of the analytical system itself.^15^, ^16^ At the Milan Conference in Europe in 2014, the European Federation of Clinical Chemistry and Laboratory Medicine (EFLM) and other organizations described three models of performance specifications: a model based on clinical results (model 1), a model based on biological variation (model 2), and a model based on state‐of‐the‐art instrumentation (model 3).^17^ Since the data of model 1 are difficult to obtain, model 2 is widely promoted and applied by laboratories. However, the performance specifications of urinary biochemical analytes have no data about biological variation, so we selected a quality goal of urinary biochemical analytes based on model 3 in the present study. Therefore, we chose the external quality assessment (EQA) standard of China as the quality goal. The data were based on the overall urinary biochemical analyte testing capabilities of laboratories in China. The National Center for Clinical Laboratories of China has collected all the data of laboratories participating in the urinary biochemical analyte proficiency test and finally determined the quality goals (TEa) of each analyte based on the baseline of more than 80% of the laboratories passing the proficiency test.

Previous studies have shown that the six sigma model has been widely used to evaluate the analytical performance of serum biochemical markers, immunological markers, and other analytes and to guide laboratories in designing risk‐based SQC strategies and improvement measures.^18^, ^19^, ^20^, ^21^, ^22^ However, the application of six sigma models in urinary biochemical analytes is rare at present. Therefore, we aimed to use the six sigma model to evaluate the analytical performance of urinary biochemical analytes across five laboratories, design risk‐based SQC strategies and quality improvement measures, and provide more accurate and reliable analytical results for clinical application.

## 2 MATERIALS AND METHODS

### 2.1 Materials

This study was conducted in five laboratories in China, which are simply labeled Lab A, Lab B, Lab C, Lab D, and Lab E. The urinary biochemical analytes involved in this experiment included K, Na, Cl, Ca, P, GLU, urea, Crea, TP, and mALB.

All the experiments were conducted with the AU5800 biochemical analyzer (Beckman Coulter, Brea, USA) detection platform and its original supporting reagents. The internal quality control (IQC) materials were provided by Bio‐Rad Laboratories (Bio‐Rad Inc., California, USA), including the following two levels: the normal level (level 1, lot: 68581) and high level (level 2, lot: 68582). Additionally, two EQA samples that were similar to the IQC materials were selected and provided by the National Center for Clinical Laboratories of China.

### 2.2 Methods

The methods for detecting urinary biochemical analyte levels are briefly described as follows: K, Na, and Cl levels were detected using the indirect ion selective electrode method; Ca levels were detected using the azo‐arsenic III method; P levels were detected using phosphomolybdic acid colorimetry; GLU levels were detected using the hexokinase method; urea levels were detected using urease colorimetry; Crea levels were detected using the enzymatic method; TP levels were detected using the dye (pyrophenol red‐molybdate) binding method; and mALB levels were detected using the immunoturbidimetric method.

#### 2.2.1 Calculation of sigma metrics

Referring to the following formula, sigma = [TEa (%) − |bias (%)|]/CV (%), the sigma metrics of each analyte were calculated.^23^ TEa represents the quality goal chosen by the laboratory. In the present study, based on the Milan consensus and the fact that the TEa of the urinary biochemical analytes was not provided in the biological variation database, we selected the EQA standard of China as the quality goal according to model 3.^24^, ^25^, ^26^

The CV data represent the imprecision of each analyte and were derived from six consecutive months of IQC (two levels) analysis from April to October 2020. The five laboratories all adopted the same IQC scheme, and the operation steps are briefly described as follows: under normal conditions, two levels of IQC were analyzed by the instrument at the same time twice a day, and all the samples were detected in a continuous manner (two levels of IQC were analyzed before sample testing, and when all the samples had been tested, two levels of IQC were analyzed once again); the mean value of each analyte was determined by the laboratory based on actual measurement results, and the mean value provided by the kit manufacturer was used only for reference; the CVs of urinary biochemical analytes for both IQC levels were calculated based on the actual measurement results of each laboratory (Table S1).

Bias represents the trueness of each analyte, and it was determined based on EQA samples of urinary biochemical analytes in 2020.

Two EQA samples with similar analyte concentrations in the IQC materials (level 1 and level 2) were selected. The EQA report provided by the National Center for Clinical Laboratories showed that a total of 140 laboratories, including the five laboratories in this study, used the same analytical platform for the measurement of urinary biochemical analytes. The target value of the urinary biochemical analytes was derived from the average value measured by all the laboratories (n=140). Moreover, each laboratory repeatedly measured the EQA sample five times in the same batch and calculated the single percentage difference according to the following formula. In addition, the average absolute value of the above single percentage difference was defined as the bias of that analyte and used for the calculation of its sigma metrics (Table S2). The formula for calculating bias is briefly described as follows:

$$Bias_{n}=\frac{Value_{\mathrm{measured}}‐Value_{t\arg et}}{Value_{t\arg et}}\times 100\%.$$

(n=first, second, third, fourth, and fifth)

$$Bias_{\mathrm{average}}=\frac{\left|Bias_{\mathrm{first}}\right|+\left|Bias_{\sec ond}\right|+\left|Bias_{\mathrm{third}}\right|+\left|Bias_{\mathrm{fourth}}\right|+\left|Bias_{\mathrm{fifth}}\right|}{5}.$$

#### 2.2.2 Normalized sigma method decision charts for urinary biochemical analytes

The normalized sigma method decision charts were generated through the Laboratory Medicine Information Network website (www.clinet.com.cn). In this study, the analytical performance of each analyte (level 1 and level 2) of the five laboratories was visually displayed in a normalized sigma method decision chart, where the y‐axis represents the Bias/TEa (%) and the x‐axis represents the CV/TEa (%). This chart was divided into six areas by five diagonal lines. Each area represents the level of the analyte's analytical performance. The sigma values are as follows from the bottom left to the top right: sigma≥6, 6>sigma≥5, 5>sigma≥4, 4>sigma≥3, 3>sigma≥2, and sigma<2.^27^

#### 2.2.3 Designing risk‐based SQC strategies and formulating improvement measures

**FIGURE 1:**
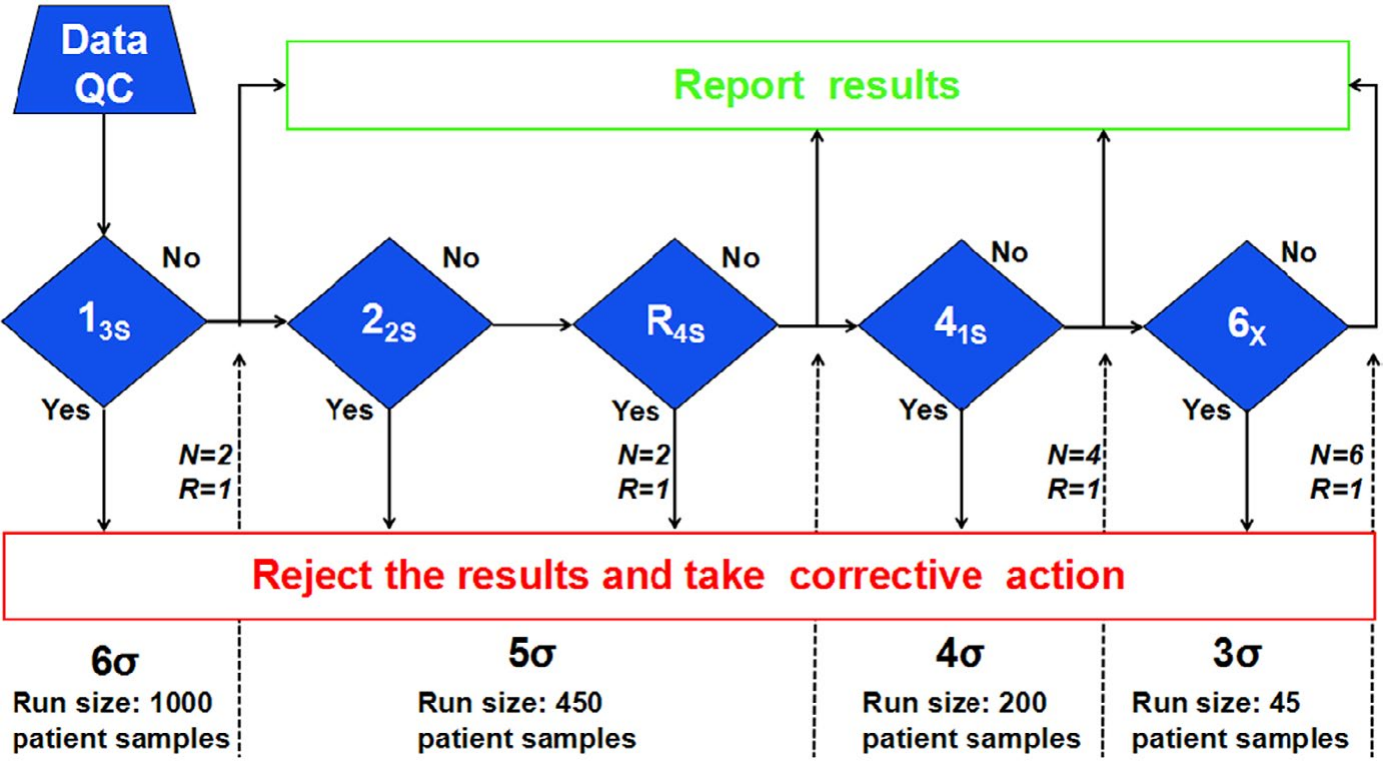
Flowchart of Westgard sigma rules with run sizes (cited from website http://www.clinet.com.cn/sigmapv/#sgm4). Sigma metric = [TEa (%) − |bias (%)|]/CV (%). First, the sigma value of each assay was calculated according to the above formula. Second, according to the sigma scale at the bottom of the flowchart, the corresponding quality control rules, the number of quality control materials (N) and the length of the analytical batch (run size) were selected. "Yes" indicates that that the quality control rules were violated, so the results were rejected and corrective measures were taken. "No" indicates that the quality control rules were not violated, so the results were accepted and reported

According to the flowchart of Westgard sigma rules, run sizes (Figure 1) and the analytical performance of urinary biochemical analytes were utilized to guide the laboratories in designing a risk‐based SQC strategy. In addition, for analytes with analytical performance less than six sigma, it was necessary to calculate the quality goal index (QGI), analyze the reasons for the observed poor performance, and prioritize formulating corresponding improvement measures. The formula for calculating the QGI was as follows: QGI=bias(%)/1.5×CV(%). When the QGI is less than 0.8, the precision is not good and needs to be improved first; when the QGI is more than 1.2, the trueness is not good and needs to be improved first; when the QGI is between 0.8 and 1.2, the precision and trueness are both poor, and corresponding improvement measures need to be taken at the same time.^27^

## 3 RESULTS

### 3.1 Sigma metrics for urinary biochemical analytes across five laboratories

**FIGURE 2:**
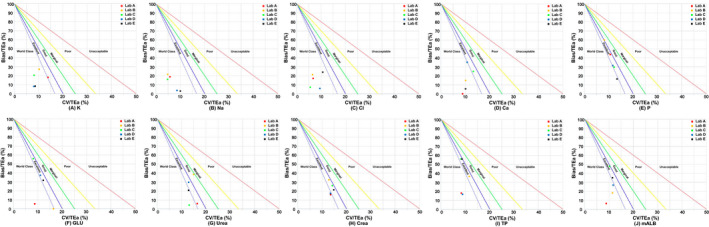
Normalized method decision charts for urinary biochemical assays using level 1 IQC material. The slopes of the five straight lines in the figures represent negative sigma values, which means that when the assay falls on one of the straight lines, the negative value of the slope represents the sigma value of the assay's analytical performance. The abscissa indicates CV/TEa (%), which represents precision. The ordinate indicates Bias/TEa (%), which represents trueness

**FIGURE 3:**
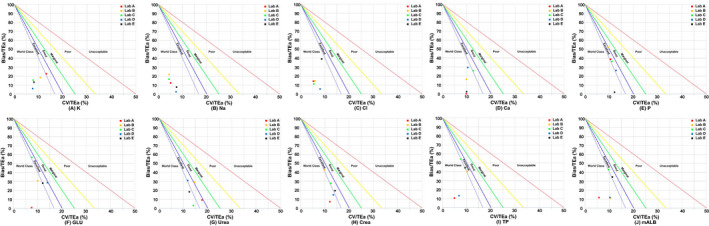
Normalized method decision charts for urinary biochemical assays using level 2 IQC material. The slopes of the five straight lines in the figures represent the negative value of sigma, which means that when the assay falls on one of the straight lines, the negative value of the slope represents the sigma value of the assay's analytical performance. The abscissa indicates CV/TEa (%), which represents precision. The ordinate indicates Bias/TEa (%), which represents trueness

In the present study, we evaluated the analytical performance of urinary biochemical analytes in five laboratories based on the six sigma model, and it was observed that when the same analyte was detected at different levels, the sigma levels were different. For example, Lab A showed a sigma level of 11.77 for urinary Cl at IQC level 1, but it was 16.43 at IQC level 2. Moreover, although this study was based on the detection of urinary biochemical analytes with the same analytical platform, its analytical performance showed significant differences among different laboratories. Taking urine urea levels as an example, the analytical performance of Lab B ranged from four sigma to five sigma, the analytical performance of Lab A and Lab D was between five sigma and six sigma, and the analytical performance of Lab C and Lab E both reached six sigma. In addition, this study also found that the analytical performance of urinary Na and Cl in the five laboratories all reached six sigma. However, the analytical performance of urinary P only reached six sigma in Lab A, while the analytical performance of other laboratories was between 4 sigma and 6 sigma. The analytical performance of urinary biochemical analytes in the five laboratories is detailed in Table 1. In addition, the analytical performance of each analyte in the five laboratories is visually displayed in normalized sigma method decision charts based on the levels of these urinary biochemical analytes (Figures 2 and 3).

**TABLE 1:** Sigma metrics of urinary biochemical analytes at two quality control levels for five laboratories

Analyte	TEa	Sigma metrics of Lab A		Sigma metrics of Lab B		Sigma Metrics of Lab C		Sigma metrics of Lab D		Sigma metrics of Lab E	
		Level 1	Level 2	Level 1	Level 2	Level 1	Level 2	Level 1	Level 2	Level 1	Level 2
K	29%	5.94	5.97	7.14	7.46	9.84	10.89	11.61	12.60	10.69	10.90
Na	26%	13.56	16.89	16.05	17.19	17.50	18.50	11.40	12.96	9.70	11.98
Cl	26%	11.77	16.43	11.54	14.04	16.31	16.37	9.91	12.03	7.04	6.90
Ca	31%	11.23	10.23	8.44	8.58	5.66	5.98	6.03	6.74	9.33	9.86
P	23%	5.14	5.77	4.77	4.98	5.71	5.73	5.90	5.71	6.16	8.13
GLU	20%	11.14	13.61	6.08	6.95	5.36	5.80	5.72	5.06	5.59	5.95
Urea	21%	5.69	5.16	4.09	4.87	7.44	7.04	5.48	5.85	6.21	6.59
Crea	17%	6.16	7.78	5.27	5.87	5.24	5.80	6.06	6.39	5.25	5.67
TP	44%	10.28	17.83	5.47	5.86	5.16	5.92	9.81	11.97	5.30	5.94
mALB	30%	10.63	14.61	7.03	8.40	5.05	5.85	6.16	8.67	5.55	5.83

Abbreviations: Ca, calcium; Cl, chloride; Crea, creatinine; GLU, glucose; K, potassium; mALB, microalbumin; Na, sodium; P, phosphorus; TEa, allowable total error, which was derived from the EQA standard of China; TP, total protein.

### 3.2 Formulation of risk‐based SQC strategies and improvement measures

In this study, risk‐based SQC strategies were designed based on a flowchart of Westgard sigma rules with run sizes (Figure 1) and the analytical performance of urinary biochemical analyte detection. For example, the sigma metrics of urinary K in Lab A were 5.94 for IQC 1 and 5.97 for IQC 2, so it was recommended that the 1_3s_/2_2s_/R_4s_ (N = 2, R = 1) multirules with a run size of 450 samples be applied as a risk‐based SQC strategy for urinary K detection. However, the analytical performance of urinary K (QC 1 and QC2) in Lab B reached six sigma; therefore, the single rule of 1_3s_ (N = 2, R = 1) with a run size of 1000 samples was recommended as a risk‐based SQC strategy for urinary K. The risk‐based SQC strategies of the urinary biochemical analytes for five laboratories are detailed in Table 2.

**TABLE 2:** Risk‐based SQC strategies of urinary biochemical assays in five laboratories

Analyte	Risk‐based SQC strategies				
	Lab A	Lab B	Lab C	Lab D	Lab E
K	1_3s_/2_2s_/R_4s_ with N=2 and R=450	1_3s_ with N=2 and R=1000	1_3s_ with N=2 and R=1000	1_3s_ with N=2 and R=1000	1_3s_ with N=2 and R=1000
Na	1_3s_ with N=2 and R=1000	1_3s_ with N=2 and R=1000	1_3s_ with N=2 and R=1000	1_3s_ with N=2 and R=1000	1_3s_ with N=2 and R=1000
Cl	1_3s_ with N=2 and R=1000	1_3s_ with N=2 and R=1000	1_3s_ with N=2 and R=1000	1_3s_ with N=2 and R=1000	1_3s_ with N=2 and R=1000
Ca	1_3s_ with N=2 and R=1000	1_3s_ with N=2 and R=1000	1_3s_/2_2s_/R_4s_ with N=2 and R=450	1_3s_ with N=2 and R=1000	1_3s_ with N=2 and R=1000
P	1_3s_/2_2s_/R_4s_ with N=2 and R=450	1_3s_/2_2s_/R_4s/_4_1s_ with N=4 and R=200	1_3s_/2_2s_/R_4s_ with N=2 and R=450	1_3s_/2_2s_/R_4s_ with N=2 and R=450	1_3s_ with N=2 and R=1000
GLU	1_3s_ with N=2 and R=1000	1_3s_ with N=2 and R=1000	1_3s_/2_2s_/R_4s_ with N=2 and R=450	1_3s_/2_2s_/R_4s_ with N=2 and R=450	1_3s_/2_2s_/R_4s_ with N=2 and R=450
Urea	1_3s_/2_2s_/R_4s_ with N=2 and R=450	1_3s_/2_2s_/R_4s/_4_1s_ with N=4 and R=200	1_3s_ with N=2 and R=1000	1_3s_/2_2s_/R_4s_ with N=2 and R=450	1_3s_ with N=2 and R=1000
Crea	1_3s_ with N=2 and R=1000	1_3s_/2_2s_/R_4s_ with N=2 and R=450	1_3s_/2_2s_/R_4s_ with N=2 and R=450	1_3s_ with N=2 and R=1000	1_3s_/2_2s_/R_4s_ with N=2 and R=450
TP	1_3s_ with N=2 and R=1000	1_3s_/2_2s_/R_4s_ with N=2 and R=450	1_3s_/2_2s_/R_4s_ with N=2 and R=450	1_3s_ with N=2 and R=1000	1_3s_/2_2s_/R_4s_ with N=2 and R=450
mALB	1_3s_ with N=2 and R=1000	1_3s_ with N=2 and R=1000	1_3s_/2_2s_/R_4s_ with N=2 and R=450	1_3s_ with N=2 and R=1000	1_3s_/2_2s_/R_4s_ with N=2 and R=450

Abbreviations: Ca, calcium; Cl, chloride; Crea, creatinine; GLU, glucose; K, potassium; mALB, microalbumin; Na, sodium; P, phosphorus; TP, total protein; N = levels of quality control; R = number of patient samples run between quality control samples. These IQC programs were the new IQC programs instituted after reviewing the sigma data.

In addition, we calculated the QGI of the urinary biochemical analytes (Sigma<6) to further determine the potential factors affecting analytical performance. Taking urinary Ca (Lab C) as an example, its QGI was 1.27 at IQC 1 and 1.47 at IQC 2, indicating that trueness was the main factor affecting its analytical performance; therefore, it is necessary to improve its trueness first. However, for urinary urea detection in Lab A, since its QGI was less than 0.8 at both IQC levels, precision improvement measures should be conducted first to improve its analytical performance. The improvement measures of the urinary biochemical analytes for the five laboratories are detailed in Table 3.

**TABLE 3:** The quality goal index and quality improvement measures for urinary biochemical assays with sigma metrics <6

Analyte	Laboratory	Sigma metrics		QGI		Improvement measures
		Level 1	Level 2	Level 1	Level 2	
K	Lab A	5.94	5.97	0.85	1.15	Imprecision and trueness
Ca	Lab C	5.66	5.98	1.21	1.47	Trueness
P	Lab A	5.14	5.77	2.61	2.44	Trueness
P	Lab B	4.77	4.98	4.76	3.68	Trueness
P	Lab C	5.71	5.73	1.61	2.22	Trueness
P	Lab D	5.90	5.71	1.79	1.36	Trueness
GLU	Lab C	5.36	5.80	4.46	4.96	Trueness
GLU	Lab D	5.72	5.06	2.25	1.35	Trueness
GLU	Lab E	5.59	5.95	1.73	1.56	Trueness
Urea	Lab A	5.69	5.16	0.23	0.35	Imprecision
Urea	Lab B	4.09	4.87	1.71	1.30	Trueness
Urea	Lab D	5.48	5.85	1.53	1.72	Trueness
Crea	Lab B	5.27	5.87	1.71	3.00	Trueness
Crea	Lab C	5.24	5.80	1.23	1.43	Trueness
Crea	Lab E	5.25	5.67	0.99	0.92	Imprecision and trueness
TP	Lab B	5.47	5.86	2.05	2.60	Trueness
TP	Lab C	5.16	5.92	4.28	4.63	Trueness
TP	Lab E	5.30	5.94	4.40	3.16	Trueness
mALB	Lab C	5.05	5.85	3.15	2.95	Trueness
mALB	Lab E	5.55	5.83	2.08	2.01	Trueness

Abbreviations: Ca, calcium; Cl, chloride; Crea, creatinine; GLU, glucose; K, potassium; mALB, microalbumin; Na, sodium; P, phosphorus; QGI, quality goal index; TP, total protein.

## 4 DISCUSSION

To the best of our knowledge, this study is the first systematic evaluation of the analytical performance of urinary biochemical analytes based on the six sigma model. The urine biochemical analytes included in this study were K, Na, Cl, Ca, P, GLU, urea, Crea, TP, and mALB, which basically cover most of the routine analytes measured in most laboratories. This was a multicenter study involving five laboratories in China, and all the experiments were conducted on the same analytical platform, which minimized the deviation caused by system differences, thereby improving the comparability of analytical results among the different laboratories.

Our study shows that the sigma values differ for different concentrations of a given urinary biochemical analyte. In other words, there is a potential relationship between the analytical performance of the analyte and its concentration. Wang et al.^28^ conducted a single‐center study to evaluate the analytical performance of urinary albumin based on sigma metrics, and the results showed that the sigma values of urinary albumin at IQC level 1 and IQC level 2 were 4.28 and 6.14, respectively. Zhou et al.^29^ applied six sigma management to evaluate the analytical performance of 16 clinical biochemical analytes. This study also indicated that the sigma values of clinical biochemical analytes were significantly different at different quality control levels. The results of these previous reports are consistent with the findings of this study, and they all completely confirm that the concentration of the analyte needs to be considered when evaluating its analytical performance based on the six sigma model.

In particular, we need to pay more attention to the role of the analyte in medical decision‐making, because its analytical performance is particularly critical for clinical diagnosis and treatment. Although this study was based on the AU5800 biochemical analyzer detection platform and its accompanying reagents, we found that the sigma value of the urinary biochemical analytes showed obvious differences among different laboratories. We preliminarily hypothesize that the possible reasons for these differences were as follows: (1) lack of consistency in performing the operating procedures by personnel among different laboratories; (2) reagent lot numbers that were not exactly the same; and (3) the potential impact of differences in temperature and humidity conditions among the laboratories on the detection system. Fasano et al.^30^ conducted a multicenter study based on the Atellica chemistry/immunoassay analyzer using sigma metrics to evaluate the analytical performance of 20 assays. This study indicated that the analytical performance of the same assay was notably different among six laboratories. In addition, Taher et al.^31^ conducted a study based on the Abbott Alinity system, applying sigma metrics to evaluate the analytical performance of 18 analytes, which indicated that individual sigma metrics varied across different laboratories. The abovementioned results were consistent with those of our study and confirmed that based on the same detection platform, the six sigma model can quantitatively evaluate and compare the analytical performance of each analyte among different laboratories. In addition, normalized sigma method decision charts were used to identify the analytical performance of each analyte among different laboratories in this study. Therefore, it was possible to visually display the analytical performance of each urinary biochemical analyte across all five laboratories.

Westgard et al.^32^, ^33^ have been committed to laboratory quality management for a long time and have successively introduced risk‐based SQC strategies, such as the Westgard multirules and Westgard sigma rules, which provide important practical bases for laboratories to adopt appropriate rules for IQC. Based on the abovementioned theory, this study developed a risk‐based SQC strategy for each laboratory. According to the flowchart of Westgard sigma rules with batch sizes and the sigma metrics for each analyte, a risk‐based SQC strategy for each urinary biochemical analyte was developed for all five laboratories. Through the implementation of this risk‐based SQC strategy, laboratories may gain certain improvements in the quality management of urinary biochemical analytes. For example, the probability for error detection might be improved or the probability for false rejection might be reduced. A study conducted by Westgard et al.^34^ indicated that the six sigma model not only helps the laboratory design an SQC strategy but also provides a good index of the risk of laboratory testing. Another single‐center study conducted by Zeng et al.^35^ also showed that a six sigma model can objectively evaluate the analytical performance of eight serum enzymes, formulate a multistage bracketed statistical quality control scheme (“start‐up” stage and “monitor” stage), and achieve the goal of reducing patient risk in real time. These previous reports and our results have completely demonstrated that the six sigma model, as a simple and practical quality management method, is very beneficial to the design of risk‐based SQC strategies in the laboratory. In addition, we also calculated the QGI of the urinary biochemical analytes (Sigma<6) to further identify the main factor that negatively impacts analytical performance, which helps a laboratory establish priorities for enhancing the laboratory's testing capabilities.

According to the QGI of the present study, we recommend that the laboratory adopt the following measures to improve the laboratory's testing capabilities: (1) standardize the performance of operating procedures by laboratory personnel and reduce experimental errors caused by human factors; (2) improve the management of the reagents to avoid the alternate use of new and old reagent lots; (3) monitor the calibration cycle of the detection system to reduce system errors; and (4) improve the maintenance of the instrument, replace old parts in a timely fashion, and improve the stability of the detection system. The research carried out by Goel et al.^36^ showed that QGI was very helpful for guiding a laboratory in determining the reasons for the poor performance of routine chemistry analyses and for guiding the adoption of appropriate improvement measures. Peng et al.^37^ also calculated the QGI of assays with sigma values less than 5 and provided clear measures for the improvement of their analytical performance. The above results are consistent with those of our study, and they completely confirmed that QGI, as an important parameter of quality improvement measures, can provide an important reference for the formulation of specific improvement plans for laboratories.

There are several limitations in this study that need to be mentioned. First, TEa, as an important parameter for evaluating the sigma value, is a prerequisite for the efficient operation of the laboratory quality system. As the biological variation data of urinary biochemical analytes cannot be obtained at present, the quality goal of this study was based on model 3 (state of the art), which is the EQA standard of China. The data were based on the statistics of the current laboratory's testing capabilities. The National Center for Clinical Laboratories of China collected the test results of all laboratories that participated in the proficiency testing activities of urinary biochemical analytes and finally determined the quality goals (TEa) of each analyte based on the baseline of more than 80% of the laboratories passing the proficiency test. Therefore, the sigma value of urinary biochemical analytes is limited by detection technology and cannot provide an objective basis from the perspective of biological variation. Second, as one of the variables of sigma metrics, certified reference materials or reference measurement procedures should be used as the first choice for evaluating the trueness of the analyte. However, for clinical laboratories, reference materials are expensive, and the feasibility of reference measurement procedures is insufficient. Therefore, referring to our previous studies,^27^, ^38^ EQA specimens and feedback data provided by the National Center for Clinical Laboratories were used to evaluate the trueness of urinary biochemical analytes. It should be noted that the target values of urinary biochemical analytes obtained in this study were not the true values but were derived from the average values calculated by all the laboratories that participated in the EQA program. Third, this was a cross‐sectional study, and there was no evaluation of the analytical performance in the five laboratories after the improvement measures were implemented, but the analytical performance of the analytes was dynamically changing. Therefore, we will continue to collect follow‐up data to provide additional reference information for the application of the six sigma model in laboratory quality management.

## 5 CONCLUSIONS

In conclusion, this is the first application of the six sigma model to quantitatively evaluate the analytical performance of urinary biochemical analytes and design risk‐based SQC strategies and quality improvement measures in five laboratories. It was confirmed that the six sigma model can be used as an important quality management tool to promote the continuous improvement of laboratory testing capabilities.

## CONFLICT OF INTEREST

The authors declare that they have no potential competing financial interests or personal relationships that could have appeared to influence the study reported in this article.

## AUTHOR CONTRIBUTIONS

QL and FMY conceived and designed the experiments. QL, GRB, XKC, JJH, YC, and MLW performed the experiments. QL and GRB analyzed the data. QL and FMY wrote the first draft of the manuscript. All the authors reviewed and edited the manuscript and approved the final version of the manuscript.

## Supporting information

Table S1‐S2

## Data Availability

Data are available on request from the authors.
