# Supplementary material for: Application of a six sigma model to evaluate the analytical performance of urinary biochemical analytes and design a risk‐based statistical quality control strategy for these assays: A multicenter study
Source: J Clin Lab Anal. 2021 Oct 15;35(11):e24059. doi: 10.1002/jcla.24059 (PMC8605169; doi:10.1002/jcla.24059)
Supplement: Supplementary file 1 — Table S1‐S2 [file JCLA-35-e24059-s001.doc]

**Supplemental Table 1** CVs of urinary biochemical analytes at two quality control levels for five laboratories (%)

| Analytes | IQC Mean | | CV of Lab A | | CV of Lab B | | CV of Lab C | | CV of Lab D | | CV of Lab E | |
| --- | --- | --- | --- | --- | --- | --- | --- | --- | --- | --- | --- | --- |
|  | Level 1 | Level 2 | Level 1 | Level 2 | Level 1 | Level 2 | Level 1 | Level 2 | Level 1 | Level 2 | Level 1 | Level 2 |
| K (mmol/L) | 30.80 | 68.30 | 4.02 | 3.77 | 2.98 | 3.17 | 2.35 | 2.26 | 2.29 | 2.17 | 2.48 | 2.33 |
| Na (mmol/L) | 80.7 | 165.0 | 1.55 | 1.34 | 1.26 | 1.17 | 1.26 | 1.17 | 2.19 | 1.96 | 2.59 | 2.01 |
| Cl (mmol/L) | 102.0 | 196.0 | 1.83 | 1.36 | 1.77 | 1.58 | 1.47 | 1.41 | 2.45 | 2.04 | 2.80 | 2.30 |
| Ca (mmol/L) | 1.74 | 2.72 | 2.76 | 3.03 | 3.13 | 3.03 | 4.15 | 3.79 | 3.34 | 3.24 | 3.14 | 3.06 |
| P (mmol/L) | 8.08 | 15.70 | 2.54 | 2.44 | 1.93 | 2.19 | 2.83 | 2.54 | 2.68 | 2.97 | 3.11 | 2.77 |
| GLU (mmol/L) | 1.57 | 16.50 | 1.70 | 1.46 | 3.29 | 2.00 | 1.66 | 1.51 | 2.20 | 2.82 | 2.44 | 2.41 |
| Urea (mmol/L) | 145 | 241 | 3.48 | 3.69 | 3.16 | 3.08 | 2.71 | 2.89 | 2.70 | 2.49 | 2.68 | 2.60 |
| Crea (mmol/L) | 5.79 | 13.80 | 2.33 | 2.04 | 2.17 | 1.64 | 2.40 | 2.14 | 2.32 | 2.28 | 2.52 | 2.41 |
| TP (mg/L) | 214 | 634 | 3.51 | 2.20 | 5.15 | 4.51 | 3.80 | 3.42 | 3.75 | 3.18 | 3.70 | 4.12 |
| mALB (mg/L) | 33.50 | 99.80 | 2.64 | 1.81 | 3.49 | 3.18 | 3.07 | 2.92 | 3.53 | 3.04 | 3.46 | 3.39 |

IQC: internal quality control; CV: coefficient of variation; K: potassium; Na: sodium; Cl: chloride; Ca: calcium; P: phosphorus; GLU: glucose; Crea: creatinine; TP: total protein; mALB: microalbumin.

**Supplemental Table 2 Bias of urinary biochemical analytes at two quality control levels for five laboratories (%)**

| Analytes | Target value | | Bias of Lab A | | Bias of Lab B | | Bias of Lab C | | Bias of Lab D | | Bias of Lab E | |
| --- | --- | --- | --- | --- | --- | --- | --- | --- | --- | --- | --- | --- |
|  | Level 1 | Level 2 | Level 1 | Level 2 | Level 1 | Level 2 | Level 1 | Level 2 | Level 1 | Level 2 | Level 1 | Level 2 |
| K (mmol/L) | 32.01 | 57.37 | 5.12 | 6.48 | 7.72 | 5.35 | 5.87 | 4.39 | 2.41 | 1.66 | 2.50 | 3.61 |
| Na (mmol/L) | 87.5 | 169.8 | 4.98 | 3.37 | 5.78 | 5.89 | 3.95 | 4.36 | 1.03 | 0.59 | 0.87 | 1.92 |
| Cl (mmol/L) | 111.3 | 186.1 | 4.46 | 3.66 | 5.57 | 3.82 | 2.03 | 2.92 | 1.71 | 1.45 | 6.29 | 10.12 |
| Ca (mmol/L) | 1.75 | 2.40 | 0.00 | 0.00 | 4.57 | 5.00 | 7.52 | 8.33 | 10.86 | 9.17 | 1.71 | 0.83 |
| P (mmol/L) | 8.05 | 10.41 | 9.94 | 8.93 | 13.79 | 12.10 | 6.83 | 8.45 | 7.20 | 6.05 | 3.85 | 0.48 |
| GLU (mmol/L) | 3.78 | 15.21 | 1.06 | 0.13 | 0.00 | 6.11 | 11.11 | 11.24 | 7.41 | 5.72 | 6.35 | 5.65 |
| Urea (mmol/L) | 161 | 233 | 1.20 | 1.96 | 8.09 | 6.01 | 0.84 | 0.64 | 6.21 | 6.44 | 4.35 | 3.86 |
| Crea (mmol/L) | 6.12 | 11.11 | 2.65 | 1.12 | 5.56 | 7.38 | 4.42 | 4.59 | 2.94 | 2.43 | 3.76 | 3.33 |
| TP (mg/L) | 291 | 712 | 7.90 | 4.78 | 15.81 | 17.56 | 24.40 | 23.74 | 7.22 | 5.92 | 24.40 | 19.52 |
| mALB (mg/L) | 21.52 | 93.58 | 1.95 | 3.55 | 5.48 | 3.29 | 14.50 | 12.91 | 8.27 | 3.65 | 10.78 | 10.24 |

K: potassium; Na: sodium; Cl: chloride; Ca: calcium; P: phosphorus; GLU: glucose; Crea: creatinine; TP: total protein; mALB: microalbumin.
